# Use of Non-Thermal Plasma as Postoperative Therapy in Anal Fistula: Clinical Experience and Results

**DOI:** 10.3390/biomedicines12081866

**Published:** 2024-08-15

**Authors:** Régulo López-Callejas, Pasquinely Salvador Velasco-García, Mario Betancourt-Ángeles, Benjamín Gonzalo Rodríguez-Méndez, Guillermo Berrones-Stringel, César Jaramillo-Martínez, Fernando Eliseo Farías-López, Antonio Mercado-Cabrera, Raúl Valencia-Alvarado

**Affiliations:** 1 Plasma Physics Laboratory, Instituto Nacional de Investigaciones Nucleares, Carretera México-Toluca S/N, La Marquesa, Ocoyoacac 52750, Mexico; regulo.lopez@inin.gob.mx (R.L.-C.); antonio.mercado@inin.gob.mx (A.M.-C.); raul.valencia@inin.gob.mx (R.V.-A.); 2 Medical Center ISSEMyM Toluca, Av. Baja velocidad 284 km. 57.5, San Jerónimo Chicahualco, Metepec 52170, Mexico; pasquinely@hotmail.com (P.S.V.-G.); mariobeta74@hotmail.com (M.B.-Á.); stringelb@hotmail.com (G.B.-S.); cesar_jara77@yahoo.com (C.J.-M.); fernandoflopez96@gmail.com (F.E.F.-L.)

**Keywords:** anal fistula, fistulectomy, fistulotomy, non-thermal plasma, wound healing

## Abstract

Anal fistula, characterized by abnormal tracts between the perianal skin and the anal canal, presents challenges in treatment because of its diversity and complexity. This study investigates the use of non-thermal plasma as a postsurgical therapy for anal fistula, aiming to promote healing and tissue regeneration. A specialized plasma reactor was designed to apply non-thermal plasma within the anorectal cavity practically. Non-thermal plasma treatment was administered to 20 patients including 10 undergoing fistulectomies and 10 undergoing fistulotomies. The average duration of non-thermal plasma application in the operating room was shorter for fistulotomies. The pain reported the day after surgery was similar in both groups. Improvements in the number of evacuations starting from the day after surgery, as well as the assessment of stool quality using the Bristol scale, indicated satisfactory intestinal recovery. Fistulotomy patients exhibited faster wound healing times. These findings underscore the efficacy of non-thermal plasma as a postoperative therapy for anal fistula, enhancing healing and recovery outcomes without increasing complication risks.

## 1. Introduction

Anal fistula represents a common pathology in anorectal surgical practice, characterized by the presence of abnormal tracts that connect an opening in the perianal skin with the anal canal. Although the majority of anal fistula in adults originates from cryptoglandular infection, its management remains a clinical challenge because of its diversity in presentation and complexity [1].

The distinction between simple and complex anal fistulas remains a topic of significant discussion [2,3,4]. Simple fistulas are generally managed with fistulotomy, which involves opening and draining the fistulous tract without causing fecal incontinence. Conversely, complex fistulas often necessitate more sophisticated treatments because of their extensive involvement and potential impact on anal sphincters [5]. Complex fistulas typically involve a considerable portion of the anal sphincter complex, often more than 25%. They may include factors such as prior fistulas in women, multiple tracts or openings, or associations with inflammatory bowel disease. The classification of anal fistulas is based on their relationship to anal sphincters, categorized into intersphincteric, trans-sphincteric, suprasphincteric, and extrasphincteric types [4,6]. Identifying the specific type of fistula within this classification system is essential for determining the optimal treatment approach and achieving favorable outcomes for patients with anal fistulas.

The main goal of surgical intervention for anal fistula is to eliminate sepsis and promote healing of the tract, preserving the sphincters and continence of the patient [7,8]. Complex anal fistula represents a particular challenge because of its high incidence of anal sphincter involvement and the consequent risk of incontinence. To prevent recurrence, it is essential to eradicate the fistulous tract completely, which requires meticulous surgical precision and a deep understanding of anal anatomy and advanced surgical techniques [9,10,11].

Accurate identification of the underlying cause of recurrence and navigation through the altered anatomy after surgery requires advanced techniques and a personalized approach. The alternatives available for the treatment of anal fistula, especially in complex and recurrent cases [12], include fistulectomy, fistulotomy, seton placement, fibrin glue injection, endorectal advancement flap, repair of autologous adipose tissue, the ligation of intersphincteric fistula tract (LIFT) technique, fistula clip closure, proximal cauterization around the internal opening, the emptying regularly of fistula tracts and curettage of tracts (PERFACT) procedure, the video-assisted anal fistula treatment (VAAFT) procedure, and fistula-tract laser closure (FiLaC^TM^) [4,13,14,15,16,17,18]. These studies have demonstrated encouraging outcomes regarding fistula closure and reduced recurrence [19]. However, more research is needed to define the optimal role of these therapeutic strategies [20,21]. Multidisciplinary collaboration among specialists is essential in offering a comprehensive and personalized approach to treat complex anorectal fistula [22,23].

Both fistulectomy and fistulotomy are widely used surgical procedures in the management of anal fistulas. Fistulectomy involves complete removal of the fistula, including surrounding infected tissue, to prevent recurrences. On the other hand, fistulotomy consists of incising and draining the fistula, leaving the wound open to heal. The choice between fistulectomy and fistulotomy depends on factors such as the location and complexity of the fistula, as well as the surgeon’s experience and preference [4,24,25].

In recent years, various sphincter preservation techniques have been proposed to minimize injuries and optimize functional results in the treatment of anal fistula [25,26,27]. However, the variety of suggested procedures, combined with a lack of follow-up data and variable clinical outcomes, has led to confusion and skepticism, limiting and restricting the utilization of these methods in actual medical settings.

Non-thermal plasma (NTP), a significant advancement in current medicine, has become a crucial tool in wound healing, redefining medical standards with its effectiveness in various areas, such as dentistry [28,29], dermatology [30], chronic wounds [31], neck tumors [32], and burns [33], to name a few. NTP, which generates reactive oxygen and nitrogen species [34], promotes healing with precision and control over damaged tissue, accelerating the healing process and minimizing scars and infections [35,36].

This study aimed to enhance medical care in the treatment of anal fistulas and explore new therapeutic perspectives. The efficacy and safety of NTP in the postsurgical treatment of anal fistulas were investigated, focusing on fistulectomy and fistulotomy surgeries. A direct comparison was made between applying NTP in both procedures to evaluate its effectiveness. This analysis considered parameters such as postoperative recovery time, pain levels, wound healing time, and potential adverse reactions including dehiscence, bleeding, infections, and other complications. A comparative assessment of fecal incontinence was also conducted among the NTP-treated groups.

## 2. Materials and Methods

### 2.1. Instrumentation

**Figure 1:**
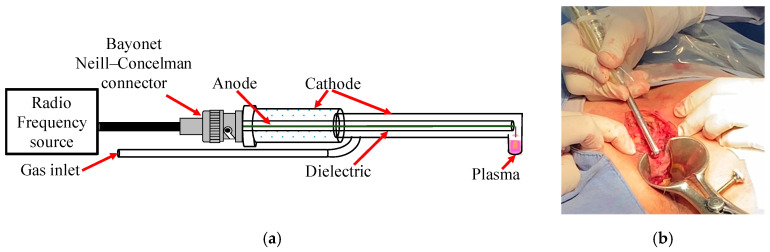
(**a**) Reactor with 90° angulation designed for use in anal fistula surgeries and (**b**) its application on tissue.

Considering our previous research on the clinical applications of NTP therapy in patients [28,29,31,32,33], necessary modifications were made to the design of the plasma reactor. First, the reactor outlet nozzle was adjusted to have an angle of 90° (Figure 1a), allowing NTP to be applied directly to the tissue walls in the deep anorectal area (Figure 1b). This adjustment facilitated the precise and practical application of NTP in these areas. In addition, the length of the reactor was extended to 25 cm to optimize treatment coverage compared with previously carried out procedures.

Helium was used as the working gas during the experimentation, maintaining a constant controlled flow of 0.5 L/min. The radiofrequency (RF) source applied a power of 20 W for plasma generation, resulting in an irradiance of 0.5 W/cm^2^ directed specifically to the tissue of the anorectal walls. It is worth mentioning that this irradiance remained within the safety limits established by the International Commission on Non-Ionizing Radiation Protection [37], whose safe threshold is 4 W/cm^2^. This guideline adherence guarantees the patient’s tissue safety and integrity during treatment. NTP was applied at a tissue distance of approximately 1–5 mm in the operating room and the medical consultation. The temperature in the tissue during NTP application was monitored in real time using infrared detection to evaluate the thermal distribution and prevent overheating. This approach allows for the applied irradiance to be adjusted. It ensures that the tissue temperature remains within safe ranges, between 28 °C and 32 °C, thus minimizing the risk of cell damage and optimizing therapeutic results. Maintaining this temperature is crucial to prevent thermal damage and preserve the integrity and functionality of the tissue.

**Figure 2:**
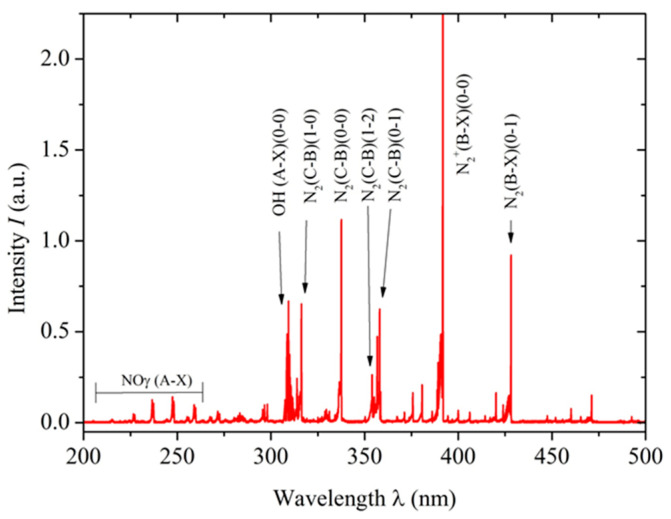
Optical emission spectrum obtained using a lambda-MinuteMan 305M monochromator, showing the molecular bands of N_2_, NOγ, and OH. The spectrum displayed covers the range from 200 to 500 nm.

The optical emission spectroscopy technique was used to detect reactive oxygen and nitrogen species generated by the non-thermal plasma. A lambda-MinuteMan 305M monochromator (Minuteman Laboratories Inc., Acton, MA, USA) in the Czerny–Tuner configuration was coupled with a Hamamatsu R955 (Shizuoka, Japan) photomultiplier, and spectral information was transmitted through a quartz optical fiber for analysis. This setup was crucial for characterizing the plasma generated by the reactor, which produced various reactive species. Figure 2 shows the resulting optical spectrum with the NTP characteristics indicated above.

Among reactive oxygen species, the hydroxyl radical (^•^OH), singlet oxygen (^1^O_2_), and hydrogen peroxide (H_2_O_2_) were identified. The presence of the hydroxyl radical in NTP may have important implications for tissue disinfection and the promotion of wound healing. Furthermore, these reactive oxygen species are known for their antimicrobial properties and ability to modulate the inflammatory response in affected tissue, further expanding NTP’s therapeutic potential in regenerative medicine. Regarding reactive nitrogen species, the γ-band phase of nitric oxide (NO), nitrogen dioxide (NO_2_), and molecular nitrogen (N_2_^+^) were detected. These species have well-documented biochemical effects, from vascular tone regulation to immune response modulation [38,39]. Particularly, NO has a significant role in wound healing, as it promotes cell proliferation and angiogenesis, which is crucial for regenerating damaged tissues. NO’s ability to regulate the inflammatory response also contributes to more effective healing. These effects demonstrate the therapeutic potential of NTP in regenerative medicine, particularly in improving wound healing in the specific context of anal fistula.

### 2.2. Patients

This study focuses on patients undergoing anorectal surgery to treat anal fistula at the ISSEMYM Medical Center. This tertiary hospital serves beneficiaries of the Government of the State of Mexico and Municipal Governments. The ISSEMYM Health Research Ethics Committee reviewed and approved the study protocol, with approval number 066/23, guaranteeing that ethical and regulatory standards were carried out. This study was carried out following national biomedical research regulations and the principles of the Declaration of Helsinki.

The inclusion criteria included adult patients (over 18 years of age) capable of granting informed consent to undergo surgery to treat anal fistula, thus ensuring homogeneity in the study group. Patients were required to have stable hemodynamic status and adequate cardiovascular function. For exclusion criteria, patients with psychiatric disorders that could affect their understanding of this study, history of epilepsy, or use of medications that affect the central nervous system were excluded. Patients with persistent medical conditions such as HIV or Crohn’s disease, as well as pregnant women, were not included in this study. Participants who opted to revoke their consent or did not proceed with further follow-up were excluded, as their inclusion could potentially skew the results and compromise the integrity of this study.

To perform anorectal surgery such as a fistulectomy or fistulotomy, meticulous preparation and positioning of the patient in the operating room were essential. Regional anesthesia included spinal anesthesia, epidural anesthesia, and nerve blocks commonly used in these anorectal procedures, providing adequate localized anesthesia with minimal side effects associated with general anesthesia. Once in the operating room, the patient was carefully placed in ventral decubitus (face down) on the operating table and was covered with sterile drapes to keep the surgical area free of contamination. Before surgery, safety measures adapted to each patient’s needs and the procedure’s complexity were applied. Once the preparation was complete, the surgeon performed a diagnostic anoscopy to locate the internal and external orifice and localization of the fistulous tract, either to remove the fistula (fistulectomy) or to open and drain it (fistulotomy), ensuring optimal visualization and manipulation of the affected area. The choice between fistulectomy and fistulotomy was based on specific clinical criteria, considering factors such as the location and extent of the fistula. Additionally, specialized postoperative care was provided to facilitate recovery and prevent complications after both anorectal surgeries.

This study applied NTP to assist in postsurgical wound healing in patients undergoing fistulectomy and fistulotomy. To this end, immediately after surgery, and with the patient still on the operating table, NTP was applied to the surgical wound. For fistulectomy cases, NTP was applied for approximately one minute for each centimeter of wound length. In cases of fistulotomy, where the wound was left open, NTP was applied for approximately three minutes for every two square centimeters of wound surface. The day after surgery, during the follow-up visit, NTP was applied again. In patients with fistulectomy, the treatment consisted of applying NTP for 30 s for each centimeter of the wound, while in patients with fistulotomy, it was applied for one minute for every two square centimeters of the wound. The plasma characteristics used in this study are detailed in Section 2.1. This protocol was designed to optimize healing and reduce recovery time.

Finally, follow-up visits were scheduled at regular intervals and adjusted according to the proximity of the patient’s residence to the hospital to monitor wound healing. The healing progress was evaluated at each visit, and NTP was applied according to the established protocol. Once the wound reached an adequate state of healing, follow-up continued through monthly appointments until completing six months. During the last visit, carried out six months postoperatively, the final state of healing was evaluated, and final analyses were carried out to determine the effectiveness of NTP treatment. This schedule of follow-up visits was designed to ensure continuous monitoring of the healing process and adapt the treatment according to each patient’s individual needs, allowing a comprehensive evaluation of the impact of NTP on postsurgical healing.

Postoperative recovery time was determined through clinical interviews and review of medical records, from the date of surgery to hospital discharge, documenting the time until the patient reached a state of recovery that allowed for discharge according to medical criteria. Pain levels were assessed with the visual analog scale (VAS), where patients rated their pain on a scale of 0 to 10 during each follow-up visit, and average scores were recorded for each surgical group. Healing time was measured by clinical inspection and photographic documentation of the wound, calculating the time from surgery to complete healing, verified by the absence of inflammation and total epithelial coverage. The Bristol scale was used to evaluate the consistency of postoperative stools, classifying them into seven types, from hard stools (type 1) to liquid stools (type 7), and providing information on intestinal function. The Wexner score was used to measure the severity of anal incontinence, with scores ranging from 0 (no incontinence) to 20 (severe incontinence), evaluating the effectiveness of treatment and its impact on each patient’s quality of life.

During this study, postoperative adverse effects were monitored in patients undergoing fistulectomies and fistulotomies. Adverse effects evaluated included dehiscence, bleeding, and infections. Bleeding was classified as early (within the first 24 h) and late (after the first 24 h). Clinical inspection, wound cultures, and evaluation of clinical signs of infection, such as erythema, heat, swelling, pain, and purulent exudate, were used to monitor surgical site infections. These adverse effects were documented during regularly scheduled follow-up visits, ensuring continuous and accurate assessment of postoperative complications.

### 2.3. Statistic Analysis

In this study, the Wilcoxon–Mann–Whitney test was used to compare the effects of NTP in fistulectomy and fistulotomy surgeries. Several parametric variables were evaluated using this test with a significance level set at 0.05. The variables analyzed included the age of the patients, the duration of surgery, the application time of the postsurgical NTP, the average application time of the NTP in consultation, the visual analog scale (VAS) to measure postoperative pain, the average number of evacuations from the day after surgery, the Bristol stool chart to evaluate the quality of evacuations, the Wexner scale for fecal incontinence, and the time to wound healing. These statistical tests were conducted to determine differences between the two treatment groups (fistulectomy and fistulotomy) in the parameters mentioned.

## 3. Results

In this study, the impact of NTP was investigated in 20 patients (see Table 1) who underwent either fistulectomy or fistulotomy for anal fistula management at the Department of General Surgery of the ISSEMyM Tertiary Hospital. The fistulectomy group consisted of ten patients, predominantly male (90%), while the fistulotomy group also included ten patients, with a majority of males (80%) and a minority of females (20%).

Within this study, we collected comprehensive data on clinical and postoperative follow-up parameters. Patients underwent either fistulectomy or fistulotomy with non-thermal plasma (NTP) as a standard part of their surgical treatments. Table 2 presents the various variables included in our data collection, encompassing specific characteristics of anal fistula, details of surgical procedures, and significant postoperative outcomes. Our assessments also covered wound healing time, pain evaluation using the specified scales, stool consistency evaluated via the Bristol scale, evacuation frequency, and Wexner fecal incontinence scale scores.

The ages of patients who underwent fistulectomy and fistulotomy for anal fistula treatment were compared. Statistical analysis (Table 2) revealed that the difference in mean ages between the fistulectomy group (51.20 ± 13.96 years) and the fistulotomy group (49.70 ± 9.48 years) was not statistically significant (*p* = 0.80).

The average operating times for fistulectomy and fistulotomy procedures were comparable. Fistulectomy had a mean duration of 33 min ± 4.83, while fistulotomy averaged 31 min (±8.43). These findings indicate that both interventions are characterized by a standard and relatively brief duration, which is advantageous for patients regarding procedural efficiency. Statistical analysis of the data showed no noteworthy variance in the duration of the operating time between the fistulectomy and fistulotomy procedures (*p* = 0.58).

The analysis of NTP application time during fistulectomy and fistulotomy surgeries revealed differences between the procedures. The mean NTP application time was notably longer in fistulectomy patients (10.90 ± 6.83 min) than those undergoing fistulotomy (4.50 ± 1.78 min). This variation is likely due to each procedure’s differing complexities and extents. Fistulotomy involves a direct, relatively short incision to open and drain the fistula, typically several centimeters long, depending on the lesion’s location and extent. In contrast, fistulectomy involves complete removal of the fistula and surrounding tissue, often requiring a larger incision, especially for more extensive lesions. The difference in surgical procedure durations may influence NTP application time. Statistical analysis comparing NTP application times between the fistulectomy and fistulotomy groups yielded a *p* = 0.03, indicating a statistically significant difference in NTP application time between the surgical procedures.

At postoperative follow-up, surgical wounds were evaluated to determine the appropriateness of NTP therapy and compare patients who underwent fistulectomy and fistulotomy. The average time of NTP application in the consultation was similar between both groups, that is, 2.68 ± 0.84 min for fistulectomy and 2.56 ± 2.05 min for fistulotomy, without showing a significant difference (*p* = 0.87). However, it varied according to the depth of the surgery in the rectum. In some cases, lidocaine administration may have influenced the duration and focus of the NTP application during the consultation.

Comparable results were seen in pain assessment using the VAS for both the fistulectomy and fistulotomy groups. The VAS scores showed a slight increase in pain levels in the fistulectomy group compared with the fistulotomy group (refer to Table 2). However, statistical analysis revealed no significant differences between the groups (*p* = 0.56), suggesting similar levels of postoperative pain following both surgical procedures for anal fistula treatment. It is worth noting that the fistulotomy group exhibited more significant variability in VAS scores, indicating varying perceptions of pain among patients.

When examining the results of the number of evacuations from the day of surgery and in the following days, differences were detected between the fistulectomy and fistulotomy groups. Patients who underwent fistulectomy had an average of 1.51 ± 0.36 evacuations, while those who underwent fistulotomy had an average of 0.82 ± 0.61 evacuations, *p* = 0.005. This statistically significant difference indicates substantial variation in the frequency of postoperative evacuations between the groups. The higher frequency of evacuations in the fistulectomy group could be linked to the complete removal of the fistulous tract and surrounding tissue, which affects postoperative evacuative function. On the other hand, the less invasive fistulotomy is associated with less alteration in intestinal function. Assessment of postoperative evacuations is crucial to understanding the impact of these procedures on evacuation function and patient well-being, highlighting the importance of considering variability in physiological responses to different surgical interventions.

Analysis of the Bristol scale revealed differences between the fistulectomy and fistulotomy groups. After fistulectomy, an improvement in the consistency of evacuations was observed, with an average score of 4.22 ± 0.60 on the Bristol scale, indicating regular or slightly firm stools. In contrast, fistulotomy resulted in a mean score of 1.93 ± 1.45 on the same scale, reflecting sausage-shaped but lumpy stools. This analysis showed a highly significant difference (*p* = 0.0002), highlighting that fistulectomy improves the quality of postoperative evacuations by promoting more formed and regular stools than fistulotomy. These findings underscore the significance of assessing the quality of postoperative defecation as a pivotal factor in evaluating the success of surgical interventions for anal fistula treatment. In summary, the frequency of evacuations seems to be more related to postoperative pain than the type of incision made. Patients who underwent fistulectomy presented higher VAS scores and more evacuations, with better consistency. In contrast, patients who underwent fistulotomy reported lower pain scores with fewer evacuations and sausage-shaped but lumpy stools.

Likewise, the postoperative results of patients who underwent fistulectomy and fistulotomy were evaluated using the Wexner score. The results showed a difference, where patients treated with fistulectomy had an average Wexner score of 1.70 ± 0.48, while those treated with fistulotomy obtained an average of 1.00 ± 0.00. This difference was statistically significant (*p* = 0.0002).

A significant difference in wound healing time was observed between patients who underwent fistulectomy and those who underwent fistulotomy for treatment of anal fistula with NTP. Patients who underwent fistulectomy showed an average wound healing time of 36.70 ± 14.87 days, while in those who underwent fistulotomy, it was 22.00 ± 13.14 days, with a highly significant difference (*p* = 0.0004). This variation may reflect the more invasive nature of fistulectomy compared with fistulotomy, which may require a more extended healing period. This analysis of wound healing time highlights the importance of considering recovery and healing when selecting the best treatment for patients with anal fistula.

Evaluation of postoperative adverse effects in our study showed that none of the patients who underwent fistulectomy and fistulotomy had complications such as dehiscence, bleeding (neither early nor late), or infections at the surgical site. This absence of complications was confirmed by extensive follow-up that included regular clinical inspections and wound cultures. The results suggest that the protocol, including NTP, was well tolerated and effective, facilitating a complication-free recovery.

**Figure 3:**
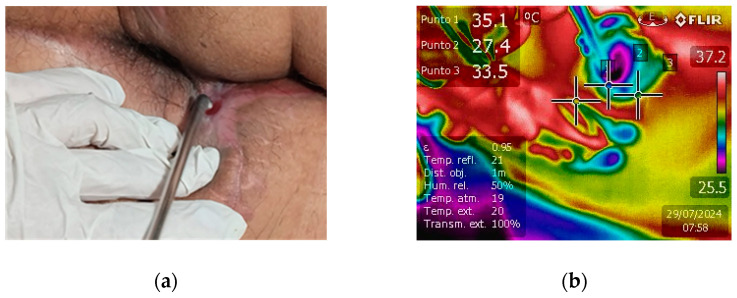
(**a**) Affected region after fistulotomy and NTP treatment. The fistula extends notably from the anal to the scrotal region, with new skin tissue formation and almost complete closure, indicating healing progress. (**b**) Real-time tissue temperature monitoring during NTP treatment.

The clinical efficacy of NTP in promoting wound healing was compellingly demonstrated in a case of extensive fistulotomy. The fistula extended markedly from the anal area towards the scrotal region, posing a significant treatment challenge. The application of NTP therapy led to exceptional healing progress, as shown in Figure 3a, which reveals nearly complete fistula closure. The formation of new skin is visible, underscoring NTP’s potential as a revolutionary treatment modality for complex and extensive wounds. This case highlights NTP’s ability to stimulate rapid tissue regeneration and provides strong evidence for its inclusion in advanced wound care protocols.

Infrared detection was used to monitor tissue temperature during real-time NTP treatment (see Figure 3b). Generally, the temperature was maintained between 28 °C and 35 °C, a range established as safe and not associated with thermal damage. This temperature range is sufficient to prevent adverse effects such as protein denaturation or cellular damage, thus guaranteeing the safety and effectiveness of the treatment. Thermography turned out to be an essential tool to ensure that NTP was applied safely, preserving the integrity of the treated tissue [39,40].

## 4. Discussion

The use of non-thermal plasma (NTP) in anorectal surgeries, such as fistulectomy and fistulotomy, has generated significant interest in the Department of General Surgery, Proctology section of the ISSEMYM Medical Center Hospital, because of its potential to improve the healing of surgical wounds. In this study, we developed an NTP-generating reactor with a 90° outlet for precise applications to the internal tissue of the rectum to improve postoperative outcomes and reduce complications associated with conventional sutures. Below, we discuss the key results obtained in this study and their clinical relevance in anorectal surgery.

In this prospective study, the impact of NTP was investigated in patients who underwent fistulectomy and fistulotomy for the treatment of anal fistula. There was an unequal proportion of male and female patients in both treatment groups, aligned with the higher incidence of anal fistula in men, according to the medical literature [4,9,41,42]. These manifestations highlight the importance of considering gender differences in evaluating and treating these conditions, given their potential impact on the effectiveness of interventions and clinical outcomes.

In this comprehensive study conducted to evaluate the clinical effects of fistulectomies and fistulotomies in selected patients, the application of NTP was included as a fundamental part of the surgical procedure. The results revealed that none of the treated patients experienced clinically significant adverse effects, such as dehiscence, bleeding, or complications, due to these procedures. During the six-month follow-up after surgery, adequate healing of the surgical wounds was observed, without infection or opening of these wounds.

The findings of this study indicate that the age of patients undergoing fistulectomy and fistulotomy for the treatment of anal fistula does not seem to have a significant impact on surgical results or postoperative recovery. Although age differences were observed between the fistulectomy and fistulotomy groups in our sample, these differences could reflect demographic and specific variations in the study population. It is important to note that, according to our statistical analysis, age did not show a significant association with the results (*p* = 0.80). Therefore, there is insufficient evidence to affirm that there is a significant difference in age between patients undergoing fistulectomy and those undergoing fistulotomy, which supports the idea that age alone does not critically influence postoperative outcomes. Our findings are consistent with prior research, which has similarly identified inconsistencies in the correlation among age, gender, and type of surgery. The complexity of this topic is underscored, and the significance of considering various factors when interpreting results and comparing them with other research is emphasized [3,4,43].

Both fistulotomy and fistulectomy are surgical procedures commonly used in the treatment of anal fistula. Our study demonstrated that the average operating times were comparable between both procedures (*p* = 0.58), supporting the effectiveness and precision of both techniques in addressing this condition. This consistency in operating times reflects the clinical effectiveness of fistulotomy and fistulectomy in the treatment of anal fistula. The findings align with studies conducted by other researchers [3,24,42,44], which underlines the reliability of these procedures in different clinical settings and supports their widespread application in medical practice.

The application time of NTP in postsurgical anal fistula wounds was observed to be significantly longer in fistulectomies compared with fistulotomies, with up to 2.5 times longer application time. This difference reflects a statistically significant association between the type of surgical procedure and the amount of NTP applied, suggesting that the extent and complexity of each surgical intervention may influence the time required for NTP application. Specifically, the wound is initially sutured in fistulectomies, and NTP is applied postsurgery. Additionally, during follow-up, the area may need to be accessed for NTP application, which can be challenging. This variation in application time underscores the need for tailoring NTP treatment based on the specific characteristics and complexities of the anal fistula. Although the statistical significance with a *p*-value of 0.03 is slightly below the conventional threshold of 0.05, the observation remains relevant, highlighting the importance of adapting the NTP treatment protocol to individual patient needs and the nature of the surgical intervention.

Our study represents a significant advance by being a pioneer in the investigation of NTP in postsurgical anal fistula. To date, the scientific literature has mainly focused on the use of NTP in the treatment of chronic wounds, such as those associated with diabetic foot [45,46,47,48,49], which highlights the originality and relevance of our work. Additionally, other research has explored the use of NTP in similar surgical contexts, such as in the treatment of venous ulcers [34,50], skin graft wounds [51,52], and dermatological procedures [53,54], thus broadening the perspective on its potential use in various clinical areas.

The existing literature on NTP treatment in chronic wounds provides valuable insights into wound healing [31,47,48,49]. However, it is essential to consider that these studies focus on chronic wounds and may differ in technical details and application protocols compared with their use in patients undergoing fistulectomy and fistulotomy for the treatment of anal fistula. Therefore, although these findings help understand the effects of NTP on wound healing, caution is required when applying them to specific surgical procedures in patients with anal fistula.

Furthermore, NTP is safe and effective in promoting wound healing in various medical fields [34,55,56]. Previous studies have demonstrated that NTP can stimulate cell regeneration and collagen production, potentially leading to improved healing outcomes in patients with anorectal fistula. The comparison of NTP with conventional techniques, such as staples or sutures for treating anorectal fistula, is an area of interest for future research. Comparative studies could help determine the relative efficacy of each technique in terms of application time, healing success rate, and potential postoperative complications. Integrating NTP in managing anorectal fistula represents a significant advancement in the quest for more effective therapeutic options. However, further research is needed to elucidate the full implications of this technique in routine clinical practice.

A comparison of postoperative pain outcomes between the fistulectomy and fistulotomy groups in patients with anal fistula treated with NTP revealed no significant differences in the pain levels experienced by the patients (*p* = 0.56). Although the average VAS scores were slightly higher in the fistulectomy group than in the fistulotomy group, this difference did not reach statistical significance, consistent with the findings reported in the previous scientific literature [8,57,58]. It is interesting to highlight the variability observed in the VAS scale scores in the fistulotomy group, characterized by a more significant standard deviation. This variability suggests a more diverse perception of pain in this group, possibly influenced by individual factors such as pain tolerance, preoperative anxiety, or previous experience with pain. Overall, pain levels were lower compared with other available treatments, which is encouraging in the context of anorectal surgical procedures [59,60,61]. These results highlight the importance of considering individual perception of pain when planning postoperative care and selecting strategies for its management in surgical procedures of this type, even when no statistically significant differences are observed among treatment groups.

After fistulectomy or fistulotomy surgery, the time for a patient to evacuate normally can vary depending on the complexity of the surgery, postoperative inflammation, and other individual factors. Generally, most patients achieve regular evacuation within the first few days, although some may experience delays due to pain or inflammation [62,63,64,65]. Our study observed that patients who underwent fistulectomy evacuated on average a day and a half after surgery, while those who underwent fistulotomy did so on average the next day. These results revealed significant differences in the frequency of postoperative evacuations between both groups (*p* = 0.005), indicating a differential impact on intestinal function associated with the nature of each surgical procedure. The clinical significance of these differences highlights the need to consider gastrointestinal implications when planning postoperative care, which may improve clinical outcomes. Prompt recovery of evacuations is crucial in these surgeries, highlighting the relevance of exploring treatments such as NTP to facilitate early and successful recovery of bowel function in patients with anal fistula.

Assessment of patients’ well-being, especially in cases of anal fistula, is essential in clinical care. The seven-point Bristol scale [66] has been instrumental in evaluating stool consistency and patient well-being. In our study, patients who underwent fistulotomy showed a notable improvement in stool consistency compared with those who underwent fistulectomy, suggesting the potential benefit of NTP on patient health [67,68]. The scientific literature reports that over 50% of cases show good fecal consistency, with a minor percentage experiencing constipation or diarrhea. In our study, only a few patients reported symptoms of diarrhea, indicating that NTP may benefit postoperative patients.

The improvement in postoperative stool quality after fistulotomy may be due to reduced local inflammation of the anus, potentially lowering the risk of fecal incontinence and improving patient quality of life.

Additionally, we evaluated the postoperative outcomes using the Wexner score. Fistulectomy patients showed a higher average Wexner score compared with those who underwent fistulotomy. This difference was statistically significant. These findings suggest that, although both procedures are effective, fistulotomy may result in better outcomes for preserving fecal continence.

Patients treated with NTP showed a longer healing time in cases of fistulectomy compared with fistulotomy. This difference may be attributed to the greater extent and complexity of the fistulectomy procedure, which involves the removal of the fistulous tract and possibly more extensive resection of tissue, which may require a more extended healing period compared with fistulotomy, which is a less complicated procedure invasive and more conservative. Other authors have observed similar results [69,70], although some studies have reported that fistulectomies heal more quickly than fistulotomies [24,42]. It is essential to highlight that during a six-month follow-up, no recurrence was observed, which underlines the long-term effectiveness of applying NTP in these surgical procedures.

Wound healing varies considerably depending on the type of surgery and the characteristics of the wound. In patients undergoing fistulectomies, which involve bloody wounds, the experience of the surgeons participating in this study has shown that postoperative pain usually exceeds 5/10 on the VAS scale, with an increase during defecation and the occasional appearance of constipation. In some cases, bleeding has been observed in the first 48 h. These factors contribute to the fact that the healing process of these wounds can extend up to 60 days on average, which is consistent with the existing literature [71,72]. In contrast, fistulotomies tend to have a faster and less painful healing process [73,74]. The literature also frequently documents complications such as dehiscence, bleeding, and infections, especially in complex surgical procedures [75,76,77,78].

## 5. Conclusions

This study conclusively demonstrates the effectiveness and safety of non-thermal plasma (NTP) in the treatment of anal fistula. The results support this therapy as an innovative and highly effective option in this clinical context. A notable decrease in postsurgery discomfort was observed, with treatment application times of just a few minutes, representing a significant patient benefit. Furthermore, defecation evaluation according to the Bristol scale revealed outstanding results. Most patients achieved a defecation level of 4 on this scale for fistulectomies, which is crucial for both postoperative recovery and daily life. All treated patients were able to defecate at least once a day after surgery and, in some cases, even on the same day of surgery. These findings suggest that using NTP improves postoperative healing of the fistulectomy and fistulotomy and positively impacts postoperative pain and bowel function. During the six-month follow-up after surgery, adequate healing of the surgical wounds was observed without any indications of infection or dehiscence. None of the treated patients experienced clinically significant adverse effects, such as dehiscence, bleeding, infections, or other complications, confirming the safety of this procedure. In summary, the results of this study support the use of NTP as an effective and safe therapy for the treatment of anal fistula, with substantial advantages in terms of decreasing postoperative pain, improving intestinal function, and the absence of adverse effects. This therapeutic approach represents a promising option to increase the well-being of patients experiencing this condition.

## Figures and Tables

**Table 1:** Patients who underwent fistulectomy and fistulotomy.

Gender	Group		Total
	Fistulectomy	Fistulotomy	
Male	9 (90%)	8 (80%)	17 (85%)
Female	1 (10%)	2 (20%)	3 (15%)
Total	10 (100%)	10 (100%)	20 (100%)

**Table 2:** Results of fistulectomy and fistulotomy surgeries.

Parameter	Group		*p*
	Fistulectomy	Fistulotomy	
N	10	10	
Age	51.20 ± 13.96	49.70 ± 9.48	0.80
Operating time [minutes]	33.00 ± 4.83	31.00 ± 8.43	0.58
Postsurgical NTP application time [minutes]	10.90 ± 6.83	4.50 ± 1.78	0.03
Average NTP application time in consultation [minutes]	2.68 ± 0.84	2.56 ± 2.05	0.87
Visual analog scale (VAS)	2.82 ± 0.81	2.41 ± 1.84	0.56
The average number of evacuations starting from the day after surgery	1.51 ± 0.36	0.82 ± 0.61	0.005
Bristol stool chart	4.22 ± 0.60	1.93 ± 1.45	0.0002
Wexner score	1.70 ± 0.48	1.00 ± 0.00	0.0002
Wound healing time [days]	36.70 ± 14.87	22.00 ± 13.14	0.0004
Adverse effects	0	0	-

## Data Availability

The original contributions presented in the study are included in the article, further inquiries can be directed to the corresponding author.
